# Safety and feasibility assessment of extending the flushing interval in totally implantable venous access port flushing during the non-treatment stage for patients with breast cancer

**DOI:** 10.3389/fonc.2022.1021488

**Published:** 2022-12-02

**Authors:** Yinhuan Wang, Hao Tian, Xianchun Chen, Jiasi Zhang, Li Wang, Haiyan Fan, Yi Zhang, Xiaowei Qi, Shaoyi Hu, Ying Yang

**Affiliations:** ^1^ Department of Breast and Thyroid Surgery, Southwest Hospital, Army Medical University, Chongqing, China; ^2^ Department of Hematology, Southwest Hospital, Army Medical University, Chongqing, China; ^3^ Department of Nursing, Southwest Hospital, Army Medical University, Chongqing, China

**Keywords:** totally implantable venous access port, breast cancer, flushing interval, thrombosis, infection, catheter obstruction

## Abstract

**Aim:**

To investigate the safety and feasibility of extending the flushing interval for the totally implantable venous access port (TIVAP) during the non-treatment stage in patients with breast cancer (BC) by retrospectively analyzing the patients’ clinical data, including the incidence of TIVAP-related complications.

**Methods:**

This single-center retrospective study included patients with BC who underwent TIVAP implantation at our hospital between January 2018 and March 2021 during their non-treatment phase and visited the hospital regularly for TIVAP flushing. Among the 1013 patients with BC who received TIVAP implantation, 617 patients were finally included on the basis of the inclusion and exclusion criteria and divided into three groups according to the length of the flushing interval: group 1 (≤30 days, n = 79), group 2 (31–90 days, n = 66), and group 3 (91–120 days, n = 472). The basic characteristics of patients in each group and the incidence of TIVAP-related complications (catheter obstruction, infection, and thrombosis) were analyzed.

**Results:**

No significant intergroup differences were observed in age, body mass index (BMI), tumor stage, pathological staging, implantation approach, chemotherapy regimen, duration of treatment, and TIVAP-related blood return rate (P > 0.05). Among patients from all three groups, 11 cases of catheter pump-back without blood and eight cases of TIVAP-related complications such as infection, thrombosis, and catheter obstruction were recorded. However, no significant differences in TIVAP-related complications were observed among the three groups (P > 0.05).

**Conclusion:**

Extending the TIVAP flushing interval beyond three months during the non-treatment stage in BC patients is safe and feasible and did not increase the incidence of TIVAP-related complications.

## Introduction

According to the survey data from the International Agency for Research on Cancer (IARC), breast cancer (BC) accounted for 11.7% of all new cancer cases worldwide in 2020, and showed the highest incidence among all cancers (1). Six to eight cycles of chemotherapy over a total treatment period of 4-6 months are essential for systemic treatment in BC. For patients who require targeted therapy, the duration of treatment may be as long as a year. Notably, patients with advanced BC often require long-term maintenance therapy because of the erratic changes in their conditions, and their treatment duration may exceed one year (2).

Chemotherapeutic drugs are categorized into vesicant, irritant, and non-vesicant agents on the basis of the degree of vascular irritation (3). For example, the anthracyclines commonly used in BC chemotherapy, such as pirarubicin and epirubicin, are vesicant agents, and extravasation of these agents may cause tissue necrosis, tissue dysfunction, and permanent disfigurement of the port (4). Therefore, selection of the ideal vascular access that can be retained over long periods of time and does not adversely affect the vessels for BC chemotherapy is particularly important. A totally implantable venous access port (TIVAP) primarily consists of an injection seat for puncture and an intravenous catheter, and is suitable for infusion of high-concentration chemotherapy drugs and nutritional support therapy (5). In comparison with other central venous access devices such as the central venous catheter (CVC) and peripherally inserted central catheter (PICC), the catheter in TIVAP is unexposed on the body surface, making it convenient for patients’ routine life. Furthermore, since it is implanted deep in the body, it shows a low incidence of infection and is associated with a higher degree of safety, significantly improving the patient’s quality of life (6).

TIVAP has become the preferred intravenous infusion tool for patients with BC receiving chemotherapy. For patients at advanced stages or with a high risk of postoperative recurrence, retention of TIVAP during the non-treatment period, i.e., after the end of treatment, has been suggested, considering the need for retreatment in the event of disease recurrence. Although a seven-year retrospective study investigating 204 cancer patients suggested that long-term indwelling of TIVAPs is safe and cost-effective (7), retention of TIVAPs may cause complications such as infections, thrombosis, and catheter occlusion in some cases. Therefore, regular TIVAP flushing is essential to reduce the incidence of complications and maintain the functional integrity of the device.

The European Society of Medical Oncology (ESMO) guidelines recommend saline flushing and heparin sealing at four-week intervals in cases with insufficient scientific indications (8). The manufacturer’s instructions also recommend flushing the port every four weeks during the non-treatment stage (9), and this interval is widely adopted in current clinical practice. Nevertheless, the Infusion Nursing Society (INS) standards of practice for intravenous therapy (10) provide no definite recommendation for the optimal TIVAP flushing interval. Consequently, the optimal TIVAP flushing interval is debatable due to insufficient evidence.

Furthermore, existing studies have provided inconsistent findings supporting extended TIVAP flushing intervals during non-treatment periods (11). Several studies have reported that extended flushing intervals of more than 45 days, six weeks, eight weeks, three months, and four months are safe and feasible (12–16). However, most of the previous studies recommending short (e.g., 45 days, 6 weeks) and long flushing intervals (e.g., 3 and 4 months) have drawn their conclusions on the basis of small sample sizes consisting of patients with different tumor types and different disease states. Sang-Bo (17) conducted a retrospective analysis of 154 patients with colorectal cancer who underwent TIVAP-based chemotherapy, and their findings suggested that extending the flushing interval to three months is safe. Most of the research on extending the TIVAP flushing interval has focused on patients with gastrointestinal tumors, lung cancer, gynecologic tumors, and hematologic malignancies, with only a few studies exclusively focusing on extending the TIVAP flushing interval in patients with BC. Therefore, the optimal flushing interval for maintaining TIVAP patency during the non-treatment stage in patients with BC patients is still unclear.

Most of the existing BC-based TIVAP studies recommend flushing every 4 weeks (18, 19). However, almost all BC patients are recommended to be followed up at 3-month intervals during the first two years after surgery and at 6-month intervals during the third to fifth year (20). In this scenario, flushing intervals of 45 days, 6 or 8 weeks, and 4 months would require patients with TIVAP to visit the hospital or clinic specifically for TIVAP maintenance in addition to regular follow-up visits, undoubtedly increasing the inconvenience in patients’ routine life. The extension of the TIVAP flushing interval to three months in patients with BC would make it consistent with the periodicity of follow-up visits. Therefore, on the basis of the aforementioned evidence, we designed this study to review the differences, especially those related to complications, among patients who underwent TIVAP flushing at different intervals.

In this study, we conducted a retrospective analysis to explore the adverse events and complications associated with different TIVAP flushing intervals during the non-treatment stage in patients with BC. The current guidelines recommend a TIVAP flushing interval of approximately three months for most patients, which can facilitate TIVAP maintenance, although some patients voluntarily choose flushing intervals of one to two months. The incidence of TIVAP-related complications and TIVAP dysfunction under different flushing intervals was recorded to investigate the safety and feasibility of extending the TIVAP flushing interval.

## Material and methods

### Study design

We conducted a single-center retrospective study to investigate the safety and feasibility of extending the TIVAP flushing interval during the non-treatment stage in patients with BC. Patients were grouped by flushing interval, and all baseline characteristics and TIVAP-related complications were collected and analyzed. All the TIVAPs were maintained and flushed by a clinical specialist nurse at our center. The Ethics Committee of the First Hospital of the Army Medical University approved this study, which was conducted in accordance with the Declaration of Helsinki (approval number: (B)KY202253).

### Patients and selection criteria

This study reviewed 1013 consecutive female patients with BC who underwent TIVAP implantation at the Department of Breast and Thyroid Surgery at the First Hospital of the Army Medical University between January 2018 and March 2021. The inclusion criteria were as follows: (1) patients with BC who underwent TIVAP implantation at our department and returned to our outpatient clinic for TIVAP flushing after systemic treatment; (2) patients with BC whose TIVAP was retained for more than one year; and (3) patients with BC who survived for more than one year. The exclusion criteria were as follows: (1) patients with BC who were receiving treatment for cancer recurrence, disease progression (salvage chemotherapy), or targeted therapy; and (2) patients with BC who violated the TIVAP flushing interval (unscheduled TIVAP maintenance).

**Figure 1:**
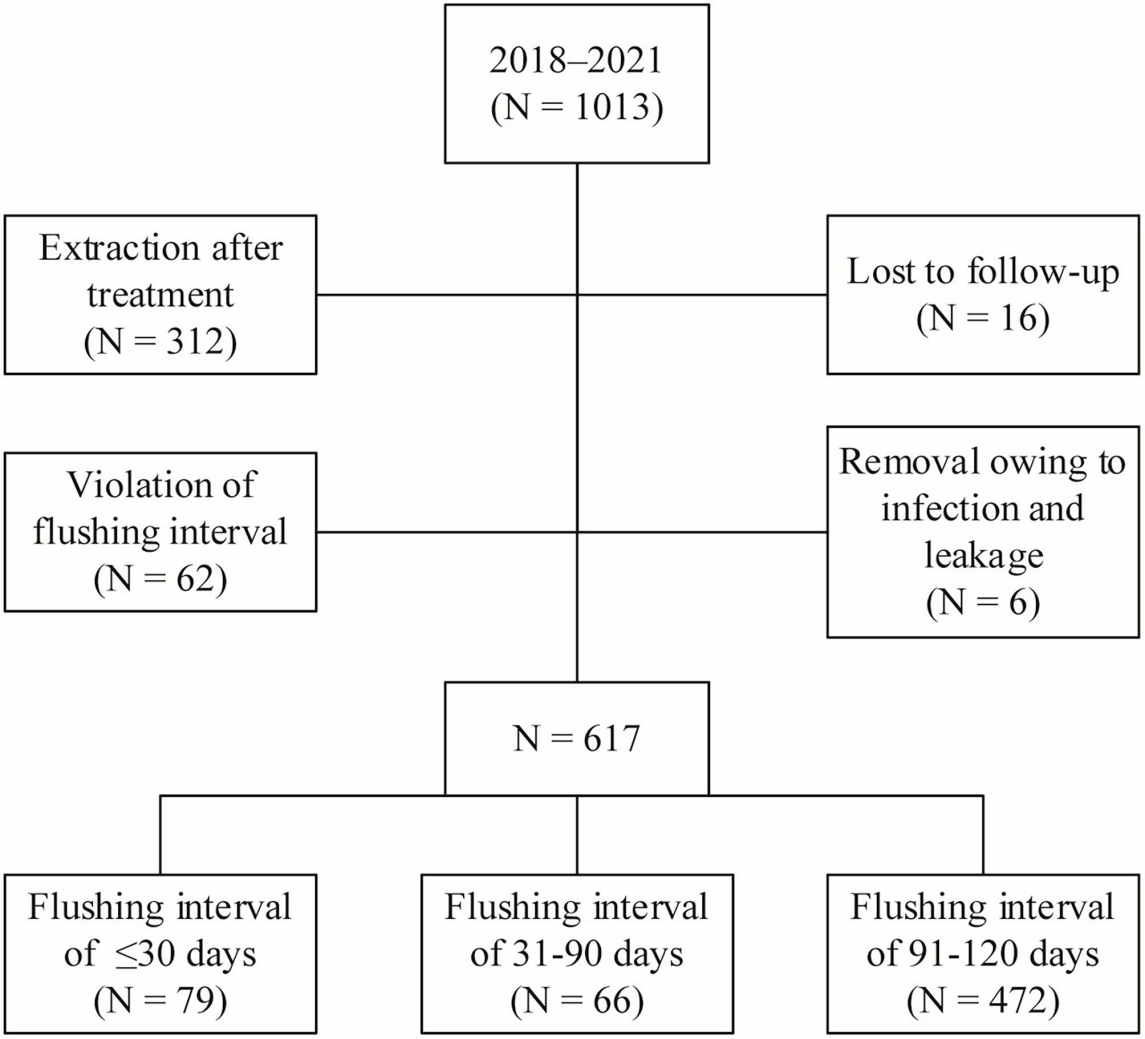
Flow chart of the study design.

A total of 396 patients were excluded after implementing the inclusion and exclusion criteria. Among them, 312 patients had undergone TIVAP removal after treatment, 16 were lost during the follow-up, 62 did not return to the hospital for flushing on time for various reasons and did not undergo TIVAP flushing at the set interval (including 28, 19, and 15 patients with a flushing interval of more than 4, 5, and 6 months, respectively), and six underwent TIVAP removal during treatment due to complications such as local infection and leakage. Finally, 617 patients whose TIVAPs were retained for more than one year were included in this study and divided into three groups according to TIVAP flushing interval: group 1 (≤30 days, n = 79), group 2 (31–90 days, n = 66), and group 3 (91–120 days, n = 472) (**Figure 1**).

### Data collection

Using the inpatient information system, we extracted the data of breast cancer patients who underwent TIVAP implantation, including information regarding their age, BMI, cancer stage, molecular subtype, chemotherapy regimen, and implantation access. Information about the flushing interval, TIVAP-related complications, and the time of removal of TIVAP was collected from the outpatient information system.

Data for TIVAP-related complications in this study, including infection, thrombosis, and catheter occlusion, were obtained from the electronic medical records and validated by additional telephone follow-ups conducted for this study. Infection in this study included local and systemic infections, and all systemic infections were confirmed by bacterial culture. Local infection was defined as an infection presenting at the port pocket and/or subcutaneous tunnel with typical manifestations such as redness, swelling, exudation, and suppuration. Systemic infection, such as catheter-related bacteremia, was defined as typical general symptoms (chill and fever) with an elevated white blood cell count before or after flushing. Catheter-related thrombosis was confirmed by Doppler ultrasonography during the follow-up examination. Catheter occlusion was categorized as partial occlusion, in which fluid could be freely infused but blood could not be freely withdrawn, and complete occlusion, in which the catheter could not be flushed and blood could not be withdrawn. All data in this study were entered and checked and analyzed by two researchers to ensure the accuracy of the results.

### TIVAP implantation

All TIVAP implantations were performed in the day surgery unit, and all surgical procedures were conducted with aseptic technique under non-invasive monitoring. Venous access was achieved through the internal jugular vein, subclavian vein, upper arm basilic vein, or median cubital vein, and all patients in this study underwent implantation of a 5-Fr/6.5-Fr single-lumen polyurethane catheter (Braun Melsungen AG, Chasseneuil, France). The implantation was performed by an experienced surgeon through the internal jugular vein and subclavian vein using ultrasound-guided venous access. Implantation through the upper arm basilic and the brachial vein was performed by a surgeon and a specialist nurse qualified in PICC implantation by using the Sedinger technique under ultrasound guidance. The catheter was implanted after ultrasound exploration of the vessels and was electrically positioned within the lumen intraoperatively. The catheter tip was located in the lower 1/3rd segment of the superior vena cava, close to the junction of the superior vena cava and the right atrium (CAJ). The surgeon administered local anesthesia to the patient and placed the TIVAP in a port pocket, followed by skin suturing and sterile dressing. After TIVAP implantation, the patient’s chest radiograph was obtained to determine if the catheter tip was placed in the desired position.

### TIVAP flushing

TIVAP flushing was conducted in accordance with the standard protocol for intravenous therapy in an outpatient clinic dedicated to intravenous therapy. The protocol consisted of four steps: (1) TIVAP assessment before flushing, which included checking the patient’s limb movement, pain, swelling of the limb, and swelling of the neck and face on the side of placement. In addition, the skin around the TIVAP was checked for pressure pain, redness, swelling, rupture, and other infection symptoms. The specialist nurse palpated the TIVAP to check port rollover and dislodgement at the connection with the catheter. (2) Disinfection of a skin area greater than 10 cm ×12 cm was followed by spreading a sterile fenestrated drape, both of which were performed after wearing sterile gloves. (3) Aspiration of 10 mL of saline was performed with a 10-mL syringe or by directly connecting the atraumatic suture needle with a 10-mL pre-flushing injection solution. A puncture was performed after deflation. Subsequently, pump-back was performed to observe unobstructed blood return, and pulse flushing was carried out after confirming the absence of abnormalities. Subsequently, positive-pressure catheter sealing was performed using 5 mL of sodium heparin diluent (100 U/mL). (4) The connecting atraumatic suture needle was removed, and the puncture site was pressed with a sterile dressing and secured with a transparent dressing. The patient was instructed to keep the dressing in place for at least 24 h. The physician also assessed catheter function and recorded the incidence of complications (catheter obstruction, infection, and thrombosis) at each flushing or patient visit.

### Statistical analysis

Data were collected and analyzed using Microsoft Excel 2019 (Microsoft Corp., Redmond, WA) and SPSS 26.0 statistical software (IBM, Armonk, NY, USA). Enumeration data are expressed as the number of cases and percentages (%), and the chi-square test or Fisher’s exact test was used for comparisons between groups. Measurement data were analyzed by one-way analysis of variance (ANOVA) or non-parametric test. P < 0.05 indicated a significant difference.

## Results

### Baseline characteristics

Data from 1013 patients with BC who underwent TIVAP implantation, preoperative neoadjuvant chemotherapy, and/or postoperative adjuvant chemotherapy from January 2018 to March 2021 were analyzed. A retrospective statistical analysis of the TIVAP flushing interval of the 617 patients, who were selected after implementing the inclusion and exclusion criteria, completed treatment, and were in the non-treatment period was performed (**Table 1**). The mean age of the patients was 47.56 ± 8.74 years (range, 25–71 years). The implantation was performed through the internal jugular vein, subclavian vein, or upper arm vein, with implantation through the upper arm vein predominating in all groups: group 1 (72/79, 91.14%), group 2 (62/66, 93.94%), and group 3 (434/472, 91.95%). The most common chemotherapy regimen consisted of eight cycles of AC(EC)-T/THP, and the application percentages in the groups were as follows: group 1 (51/79, 64.56%), group 2 (41/66, 62.12%), and group 3 (276/472, 58.50%). No significant differences were recorded in age, BMI, tumor stage, pathological type, implantation access, chemotherapy regimen, and duration of treatment among the three groups.

**Table 1:** Baseline information of patients who underwent TIVAP implantation.

Variables	Group1	Group2	Group3	P
	(n = 79)	(n = 66)	(n = 472)
***Age (years)***	47.64±8.03	46.06±8.78	48.12±9.20	0.263^a^
***BMI (kg/m^2^)***	23.72±2.77	23.44±2.35	23.48±2.86	0.577^a^
***Molecular subtype***				
Luminal A	12 (15.19)	15 (22.73)	79 (16.74)	0.602^b^
Luminal B (HR2-negative)	17 (21.52)	7 (10.61)	90 (19.07)	
Luminal B (HER2- positive)	15 (18.99)	11 (16.67)	80 (16.95)	
Triple-negative	19 (24.05)	16 (24.24)	123 (26.06)	
HER-2 Overexpression	16 (20.25)	17 (25.76)	100 (21.19)	
***Tumor stage***				
Stage I	15 (18.99)	15 (22.73)	92 (19.49)	0.927^c^
Stage II	44 (55.70)	31 (46.97)	269 (56.99)	
Stage III	9 (11.39)	12 (18.18)	51 (10.81)	
Stage IV	11 (13.92)	8 (12.12)	60 (12.71)	
***Implantation access***				
Internal jugular vein	4 (5.06)	3 (4.55)	26 (5.51)	0.926^b^
Subclavian vein	3 (3.80)	1 (1.52)	12 (2.54)	
Upper arm vein	72 (91.14)	62 (93.94)	434 (91.95)	
***Chemotherapy regimen***				
TA/TC/TAC/TCbH/TL/TLHP: 6 cycles	26 (32.91)	22 (33.33)	174 (36.90)	0.845^b^
AC (EC)—T/THP: 8 cycles	51 (64.56)	41 (62.12)	276 (58.50)	
Other regimens	2 (2.53)	3 (4.55)	22 (4.70)	

Data are presented as n (%);

a: ANOVA test; b: χ2 test; c: rank-sum test.

Chemotherapy regimen: T: docetaxel/albumin paclitaxel; A: doxorubicin; C: cyclophosphamide; L: lobaplatin; H: trastuzumab; P: pertuzumab.

### TIVAP flushing intervals of the three groups

**Figure 2:**
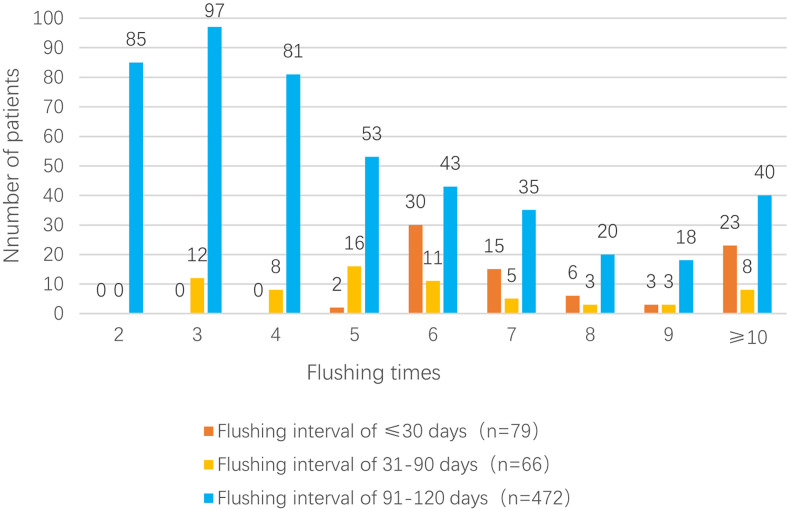
TIVAP flushing interval in the three groups of patients.

The mean flushing interval in group 1 (n = 79; ≤ 30 days) was 29.42 ± 0.39 days (median: 29.45 days), and the mean number of flushes in this group was 8.84 ± 4.18 (median: 7); among the 79 patients, 23 patients (29.1%) underwent ≥10 flushes. The mean flushing interval in group 2 (n = 66; 31–90 days) was 71.79 ± 11.67 days (median: 64.66 days), and the mean number of flushes was 6.00 ± 2.84 (median: 5); among the 66 patients, eight patients (12.1%) underwent ≥10 flushes. Finally, the mean flushing interval in group 3 (n = 472; 91–120 days) was 96.74 ± 5.33 days (median: 95.00 days), and the mean number of flushes was 4.90 ± 2.67 (median: 4); among the 472 patients, 40 patients (8.5%) underwent ≥10 flushes **Figure 2**.

### TIVAP-related blood return rate

Among the 617 patients who underwent TIVAP flushing during the non-treatment phase, no blood return was observed after catheter pump-back in 11 patients (one patient in group 1, upper arm vein group; two patients in group 2, upper arm vein group; seven patients in group 3, including one in the internal jugular vein group, one in the subclavian vein group, and five in the upper arm vein group). The difference in the blood return rate among the three implantation access groups was not significant (P > 0.05). The three groups also showed no significant differences in TIVAP-related blood return rate (P > 0.05; **Table 2**).

**Table 2:** Comparison of TIVAP-related blood return rate among the three groups.

Implantation access	Blood return	Group 1 (n = 79)	Group 2 (n = 66)	Group 3 (n = 472)	P
Internal jugular vein	Yes	4 (100)	3 (100)	25 (96.15)	0.999
	No	0 (0)	0 (0)	1 (3.8)	
Subclavian vein	Yes	3 (100)	1(100)	11 (91.67)	0.999
	No	0(0)	0(0)	1 (8.33)	
Upper arm vein	Yes	70 (97.22)	60 (96.77)	429 (98.85)	0.194
	No	2 (2.77)	2 (3.22)	5 (1.15)	
Total	Yes	77 (97.47)	64 (96.97)	465	0.416
		2 (2.53)	2 (3.03)	-98.52	
	No			7 (1.48)	

Data are presented as n (%).

### Incidence of TIVAP-related complications

Among the 617 patients who underwent TIVAP flushing during the non-treatment phase, one case each of infection and catheter obstruction was observed in group 1 (1.27%); one case each of thrombosis and catheter obstruction was observed in group 2 (1.52%); and one case of infection (0.21%), one case of thrombosis (0.21%), and two cases of catheter obstruction (0.42%) were observed in group 3. The three groups showed no significant differences in the incidence of TIVAP-related complications (P > 0.05; **Table 3**).

**Table 3:** Comparison of incidence of TIVAP-related complications among the three groups.

Complications	Group 1 (n = 79)	Group 2 (n = 66)	Group 3 (n = 472)	P
Infection	1 (1.27)	0 (0.00)	1 (0.21)	0.415
Thrombosis	0 (0.00)	1 (1.52)	1 (0.21)	0.218
Catheter obstruction	1 (1.27)	1 (1.52)	2 (0.42)	0.236
Total	2 (2.53)	2 (3.03)	4 (0.85)	0.110

Data are presented as n (%).

## Discussion

This study retrospectively evaluated the feasibility and safety of extending the TIVAP flushing interval of patients with BC during the non-treatment phase. TIVAP is now widely used in chemotherapy and supportive care for malignancies. Yuejiao et al. (12) retrospectively analyzed the findings for 563 patients with malignant tumors whose TIVAP flushing intervals were extended beyond four weeks during the non-treatment period. They found that the complication rate in the extended group (>28 days) was not significantly different from that in the non-extended group (≤ 28 days) (p = 0.28), indicating the safety of extending the TIVAP flushing interval. Kefeli et al. (13) retrospectively analyzed patients who received chemotherapy for various malignancies (mostly gastrointestinal tract tumors); 30 of these patients underwent the standard flushing regimen (four weeks, 500 U heparin), whereas 59 underwent extended flushing intervals (six weeks, 1000 U). The one-year follow-up results indicated no infection or thrombosis in patients with extended flushing intervals, confirming that an extended flushing interval of to six weeks is safe and feasible.

A total of 14 flushing interval-related publications were included in the 2021 meta-analysis by Zhao-Yu et al. (21), which suggested that extending the flushing interval by eight weeks is safe and feasible. Six publications involving 1255 patients with various malignancies were included in the 2020 meta-analysis by Clari et al. (11), which also suggested that extending the flushing interval to four to eight weeks does not increase the incidence of TIVAP catheter-related obstruction and infection. The findings of our study are consistent with those obtained by Ignatov et al. (16), who demonstrated that extending the flushing interval to more than three months can effectively maintain TIVAP patency. Furthermore, numerous studies have reported extending the TIVAP flushing interval for more than four weeks, 45 days, six weeks, eight weeks, three months, and four months, and the findings of these studies suggested that extending the flushing interval for the corresponding time period is safe and feasible. However, an optimal TIVAP flushing interval has not yet been established. Therefore, prospective, multicenter, large-sample real-world studies are needed to validate our findings.

Thrombosis is one of the common TIVAP-related complications. TIVAP-related thrombosis is associated with surgery, infection, hospitalization, thrombogenic drug use, and the release of procoagulant substances from tumor cells in patients with malignancy (22). Venous thrombosis is usually asymptomatic or may occasionally present as pain or swelling. Kuo et al. (23) reported the findings for 73 gynecologic oncology patients whose flushing intervals were extended up to three months; among these patients, seven (7/73, 9.6%) had catheter tip-related thrombosis, with no significant difference in the incidence of thrombosis between the experimental and control groups. Heibl et al. (24) reported that among 143 patients with hematologic diseases whose flushing intervals were extended to four to six weeks, three (3/143, 2.1%) developed thromboses, including one case of deep venous thrombosis and two cases of catheter tip-related thrombosis. Ignatov et al. (16) conducted a retrospective study of 349 patients with malignant tumors who had undergone an extended flushing interval and found that eight patients developed catheter-related thrombosis, including three patients with deep venous thrombosis; the overall incidence of thrombosis in their study was 2.29%, and the difference between the experimental and control groups was not significant (P = 0.465).

Consistent with these findings, the present study included two cases of thrombosis, from groups 2 and 3, that were characterized by swelling of the affected limb and neck and subsequently confirmed by ultrasound. The patients received rivaroxaban treatment for 3 months as recommended, and the ports were finally removed (25). Our results indicated no significant difference in the incidence of thrombosis among the three groups (P = 0.22), suggesting that extension of the flushing interval did not increase the incidence of thrombosis. Notably, we did not perform routine thrombosis screening for all TIVAP patients in this study, so the incidence of asymptomatic thrombosis remains unknown.

Several studies have preliminarily explored whether an extended flushing interval increases the incidence of infection. Fornaro et al. (9) reported no significant difference in infection rate between a 4-week flushing group (2/106, 1.9%) and an 8-week flushing group (8/347, 2.3%; P = 0.80). A systematic literature analysis of four single-arm studies with a total of 925 patients (15, 26–28) reported that the incidence of infection associated with flushing at 6–12-week intervals was 1.6%. However, that study did not include a statistical analysis of the infection rate at different flushing intervals.

In this study, no significant differences were observed in the incidence of infection among the three groups (P = 0.42), and only two infectious cases manifesting as sudden-onset fever and chills were recorded. One case in group 1 was diagnosed as a *Ralstonia pickettii* infection, and the other case in group 2 was diagnosed as a coagulase-negative *Staphylococcus* infection. Both patients recovered quickly after receiving antibiotic treatment.

The source of infections attributable to intravenous catheters is mostly the microbial population in the skin around the catheter, and the most common bacteria causing TIVAP-related infections are coagulase-negative staphylococci, with *Staphylococcus epidermidis*, a common bacterial parasite of the skin and mucosa, being the predominant bacteria. Most patients develop infections in the first few months after TIVAP implantation, which may be because of surgery and chemotherapy and frequent punctures with atraumatic suture needles resulting in local skin bacterial infections (29). A low incidence of infection was observed in the present study. We hypothesized that this could be because most patients had undergone TIVAP implantation more than six months previously and had completed their treatment without many of the aforementioned risks. Overall, the results of this study and previous studies suggest that extending the flushing interval does not increase the incidence of infection.

Catheter obstruction, often defined as impaired pump-back or poor push injection, is another common TIVAP-related complication. The causes of TIVAP catheter-related obstruction can be divided into catheter- and non-catheter-related factors. Catheter-related factors include catheter compression, folding, improper end position, catheter displacement, and adherence of the catheter end to the vessel wall. Non-catheter-related factors include perfusion of nutritional drugs and drug interactions causing precipitation and obstruction. A meta-analysis (30) revealed that a TIVAP flushing interval beyond four weeks is safe, and extending the flush interval to eight weeks may not increase the incidence of catheter occlusion. Zhao-Yu et al. (21) included 1318 patients in a meta-analysis and divided them into a standard flushing group (four weeks) and an extended flushing interval group (>4 weeks); the incidence rates of catheter obstruction in these two groups were 3.7% and 4.2%, respectively (P = 0.29). In our study, no significant differences were observed in the incidence of catheter obstruction among the three groups (P = 0.24). The results of the abovementioned meta-analyses and the findings of our study indicate that extending the flushing interval does not increase the incidence of catheter occlusion, although relatively significant individual differences may be present. Jiaobo et al. (31) reported normal function of TIVAP after five years of disuse in one patient. Our center also had one patient with BC who did not undergo regular flushing. She received her first flush 24 months after her last chemotherapy due to BC recurrence, and her TIVAP remained functional.

The TIVAP should be flushed regularly to prevent the accumulation of fibrin or drugs. However, few studies have reported the effects of extended TIVAP flushing intervals on blood return. In this study, among the 617 patients who underwent TIVAP flushing during the non-treatment phase, 11 patients (1.78%) did not show blood return, while the remaining 606 patients (98.22%) showed blood return, with no significant difference in the blood return rate among the three groups (P = 0.42). Regular flushing is crucial to keep the TIVAP patent and functionally intact. However, evidence for the choice between saline and heparin flushing is unavailable. Heparin flushing is considered the most vital intervention to maintain TIVAP patency, prevent catheter obstruction, and reduce the risk of catheter-related infections (8). Several prospective studies in adult oncology patients have suggested that TIVAP maintenance with a 10-mL saline flush and a 5-mL heparin (100 U/mL) seal every three months is safe and effective (15, 28, 32). Solinas et al. (15) reported the findings for 381 patients with malignancy who received TIVAP implantation and underwent saline flushing every three months and suggested that flushing did not increase the risk of TIVAP obstruction. Rasero et al. (33) used saline to seal the catheters. However, heparin sealing was performed in most of the other studies, and it remains uncertain whether heparin is more effective than saline in sealing catheters (34, 35). In the present study, catheter sealing was performed using 5 mL of 100 U/mL sodium heparin. In a subsequent study, we aim to compare the difference between saline- and heparin-based catheter sealing and their effects on the extension of the TIVAP flushing interval.

Monthly flushing is time-consuming, inconvenient, and expensive. For patients with BC who are in the non-treatment period after treatment stabilization, doctors recommend reexamination every three months, which coincides with the TIVAP flushing interval. Therefore, extension of the TIVAP flushing interval can reduce the financial burden on patients and improve their quality of life. Furthermore, it can reduce the workload of healthcare workers and the consumption of medical supplies.

Nevertheless, this study had some limitations. First, it is a retrospective study with a relatively small sample size that may have resulted in selection bias. Therefore, we hope to design prospective, randomized controlled trials in the future to validate the findings of this study. Second, the difference between saline and heparin for catheter sealing during non-treatment TIVAP flushing and their effects on catheter obstruction were not evaluated. The catheters were sealed with heparin during all TIVAP flushes, which may have influenced our results. Finally, we may have overlooked asymptomatic intra-catheter thrombosis when assessing thrombotic complications due to the absence of routine vascular ultrasound examinations.

## Conclusion

A retrospective analysis of 617 patients with extended flushing intervals during the non-treatment period revealed that extending the flushing interval to more than three months did not increase the incidence of complications such as infection, thrombosis, and catheter obstruction in comparison with the standard flushing interval commonly used in current practice. Therefore, extending the TIVAP maintenance interval to three months or beyond is a safe and feasible approach. This extension helped improve the efficiency of TIVAP maintenance, alleviated medical resource shortages to a certain extent, and reduced patients’ visit time.

## Data availability statement

The original contributions presented in the study are included in the article/supplementary material. Further inquiries can be directed to the corresponding authors.

## Ethics statement

The studies involving human participants were reviewed and approved by Ethics Committee of the First Hospital Affiliated with the Army Medical University (Grant No.: (B) KY202253). The patients/participants provided their written informed consent to participate in this study.

## Author contributions

Conception and design: YY, XQ, SH, HT. Data collection: LW, HF, XC, and JZ. Data analysis: YW, YZ. Writing and manuscript revision: YW, HT, YY. All authors contributed to the article and approved the submitted version.

## Conflict of interest

The authors declare that the research was conducted in the absence of any commercial or financial relationships that could be construed as a potential conflict of interest.

## Publisher’s note

All claims expressed in this article are solely those of the authors and do not necessarily represent those of their affiliated organizations, or those of the publisher, the editors and the reviewers. Any product that may be evaluated in this article, or claim that may be made by its manufacturer, is not guaranteed or endorsed by the publisher.
